# Case of a Male Patient With Focal Dermal Hypoplasia (Goltz Syndrome), Esophageal Polyps, Scoliosis, and Bicuspid Aortic Valve

**DOI:** 10.7759/cureus.97595

**Published:** 2025-11-23

**Authors:** Blagovest Stoimenov, Ralitsa Pancheva, Ventsislava Pencheva, Vasil Kolev, Rozita Karakasheva, Dimitar Stoyanov, Maria Stancheva, Radoslava Tsrancheva, Svilen Donev, Diana Petrova, Ventseslav Yordanov, Antonia Vasileva, Georgi Ovnarski, Diyan Genov

**Affiliations:** 1 Propedeutics of Internal Diseases, University Multiprofile Hospital for Active Treatment (UMHAT) Alexandrovska, Medical University Sofia, Sofia, BGR; 2 Propedeuticus of Internal Medicine, Medical University Sofia, Sofia, BGR; 3 Nephrology, Clinic of Nephrology, University Multiprofile Hospital for Active Treatment (UMHAT) “St. Ivan Rilski”, Sofia, BGR; 4 Medicine, Faculty of Medicine, Medical University Sofia, Sofia, BGR

**Keywords:** bicuspid aortic valve, dermal focal hypoplasia, embryonal anomalies, genetic disorders, goltz syndrome, porcn, x-linked dominant inheritance

## Abstract

Focal dermal hypoplasia (Goltz syndrome) is an extremely rare genetic disorder characterized by specific skin manifestations and a wide range of anomalies affecting the ocular, dental, skeletal, urinary, gastrointestinal, cardiovascular, and central nervous systems. The syndrome is inherited in an X-linked dominant manner and is usually lethal in males, with adult male survivors representing only about 10% of all reported cases.​​​​​​ It has been established that mutations in the *PORCN *gene (Xp11.23), which encodes proteins with a key role in embryonic development, are responsible for this condition. Diseases associated with *PORCN *include a spectrum of highly variable multisystem disorders caused by congenital anomalies in mesodermal and ectodermal structures. Craniofacial features may include facial asymmetry, nasal notches (indentations in the nostrils), cleft lip and palate, pointed chin, and small, underdeveloped ears. Dental anomalies include hypodontia (missing teeth), enamel defects, and/or abnormally shaped teeth. Herein, we report a very rare case of a male patient (only up to 10% of male patients with Goltz syndrome survive) with clinically confirmed disease (one major and two minor criteria), admitted with anemia, after severe hematemesis. During the hospital stay, he was diagnosed with polyps of the esophagus and stomach and a bicuspid aortic valve. After clinical evaluation and gastroprotective and antihypertensive treatments, he was discharged with improved condition.

## Introduction

PORCN-related developmental disorders are linked to changes in the X chromosome. Around 90% of affected individuals are females, who usually have a heterozygous or mosaic pathogenic mutation in *PORCN*. The main group of females (approximately 95%) with *PORCN*-related disorders have a de novo (new) pathogenic mutation, and the rest (around 5%) inherit the pathogenic mutation from a parent. Around 10% of all affected individuals are males. Mainly, they are mosaic for a de novo pathogenic mutation in *PORCN *[1].

PORCN-related developmental disorders result from pathogenic variants in the X-linked *PORCN *gene. Despite its extremely rare occurrence, it is important that healthcare professionals be familiar with the clinical features of this disease because of the multidisciplinary treatment that these patients require [2]. Herein, we report a very rare case of a 43-year-old man with clinically diagnosed Goltz Syndrome (one major and two minor criteria), who was hospitalized with complaints of profuse hematemesis and diarrhea and was diagnosed after fibrogastroscopy and echocardiography with polyps of the esophagus and stomach and a bicuspid aortic valve.

## Case presentation

**Figure 1:**
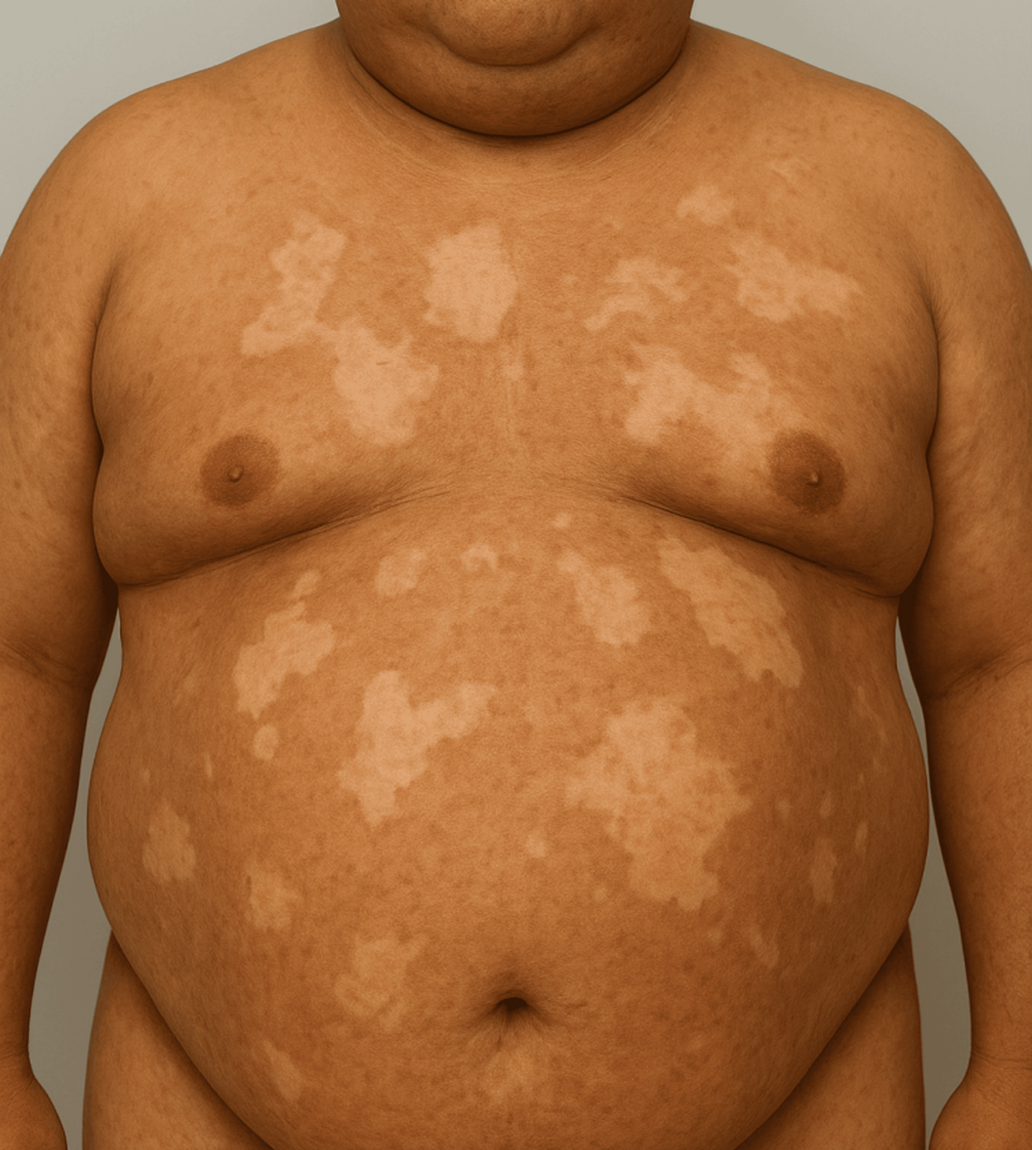
Focal dermal hypoplasia

A 43-year-old man with clinically confirmed Goltz Syndrome (one major and two minor criteria) was hospitalized in the department of internal medicine with data on profuse hematemesis and diarrhea. As a child of 14 years, he was diagnosed with basal cell carcinoma (major criteria), which was surgically removed. The gold standard for the diagnosis of the Goltz syndrome is genetic testing for the *PORCN *gene. These data were obtained from the patient's companion, as the patient had a mild intellectual developmental delay and behavior problems. From the physical examination of the patient, it was found that he had elevated blood pressure, pronounced skin atrophy, and hypopigmented spots of various shapes and sizes on the torso (Fig. 1) [3]. 

**Figure 2:**
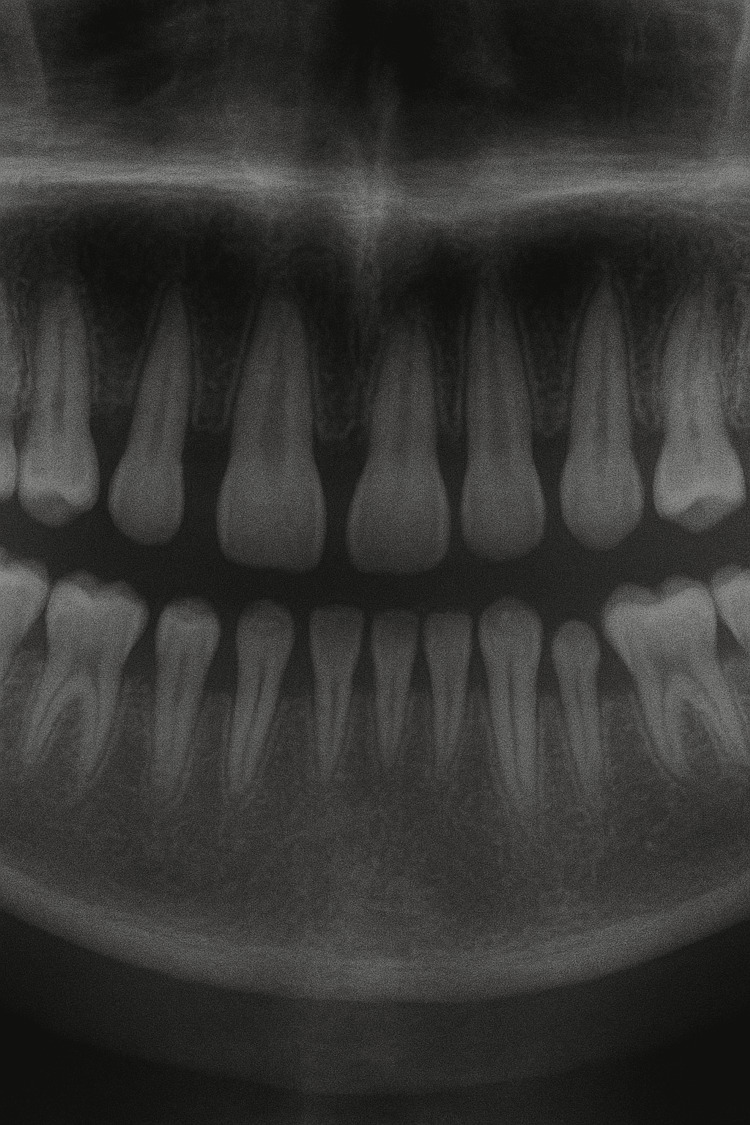
Hypodontia with small teeth roots

The patient had facial asymmetry with increased head circumference (minor criteria); the ears were low-set, microphthalmia was present, and examination of the oral cavity revealed hypodontia and short tooth roots (Fig. 2). 

**Figure 3:**
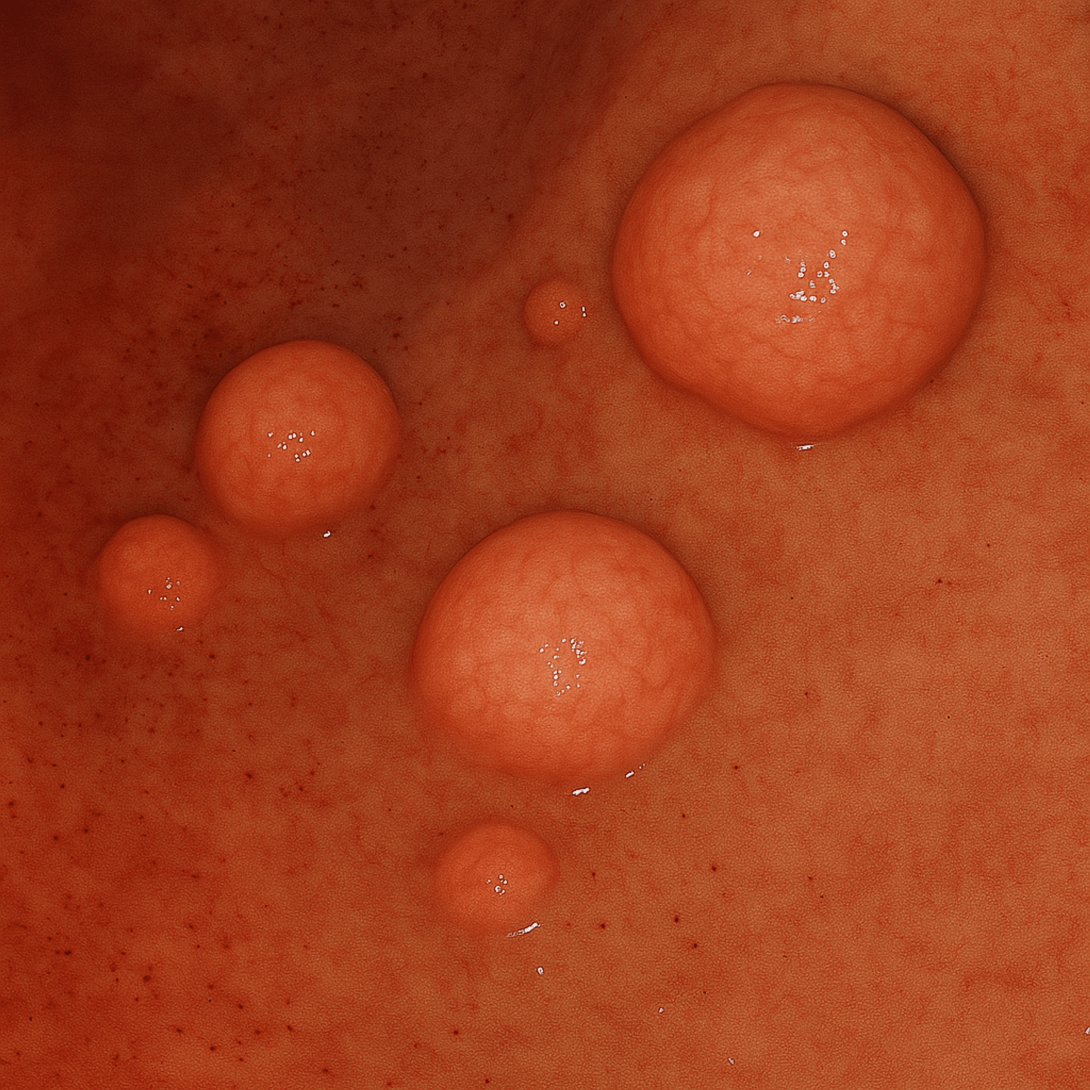
Gastroscopy showing polyps of the esophagus

The man suffered from patchy alopecia of the scalp. Different diagnostic laboratory and instrumental tests were done in order to clarify the diagnosis. Тhe patient had data on microcytic hypochromic anemia with hemoglobin levels 98 g/l, which did not require blood transfusion, elevated creatinine (134 μmol/L), and uric acid (534 mmol/l), and low potassium level (3.4 mmol/l). By fibergastroscopy, the following were found: the patient’s esophagus had pink mucosa, diffusely covered with polyps of different calibers of 5-20 mm (Fig. 3 )

**Figure 4:**
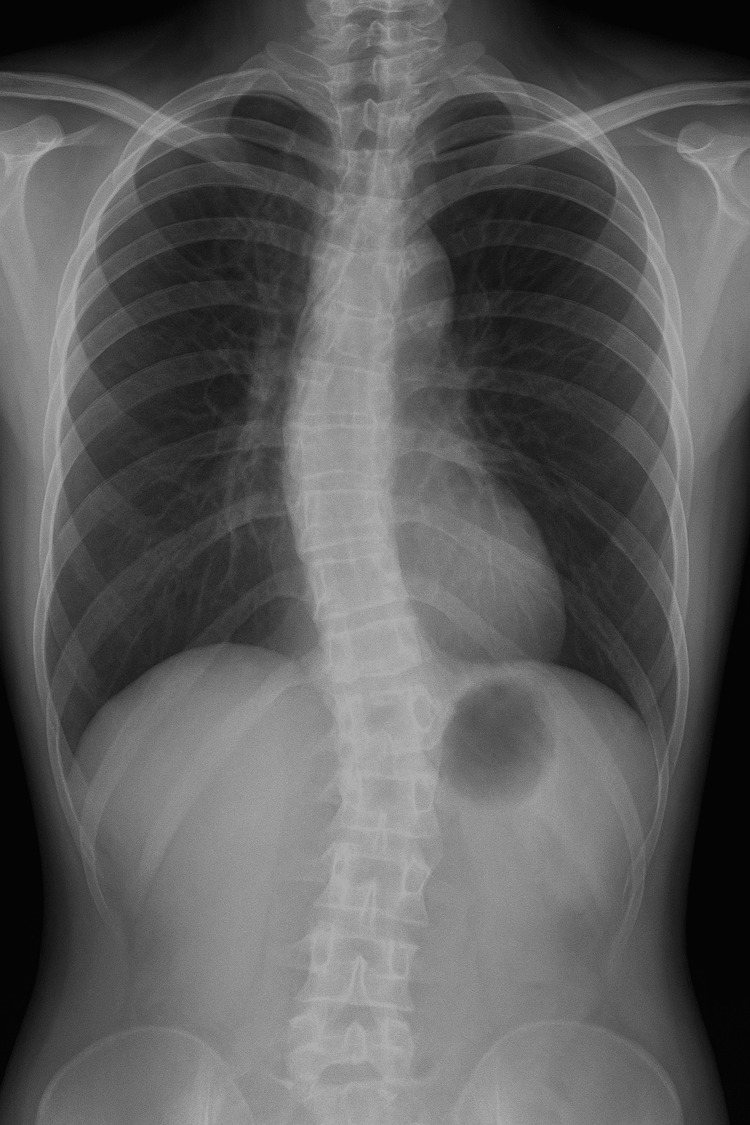
X-ray showing S-shaped scoliosis

The stomach was normal, had preserved tone and edematous hyperemic mucosa, with polyps in the fundus. The duodenum was leaky and had a normal mucosa, while the bulbus duodenoi had a hyperemic mucosa. In the liver, hypoechoic rounded areas and a diffuse process were visible during abdominal ultrasound. On chest and heart radiography, the patient had S-shaped scoliosis (minor criteria), due to which the cardiovascular shadow was superimposed on the middle and lower left lung fields (Fig. 4).

**Figure 5:**
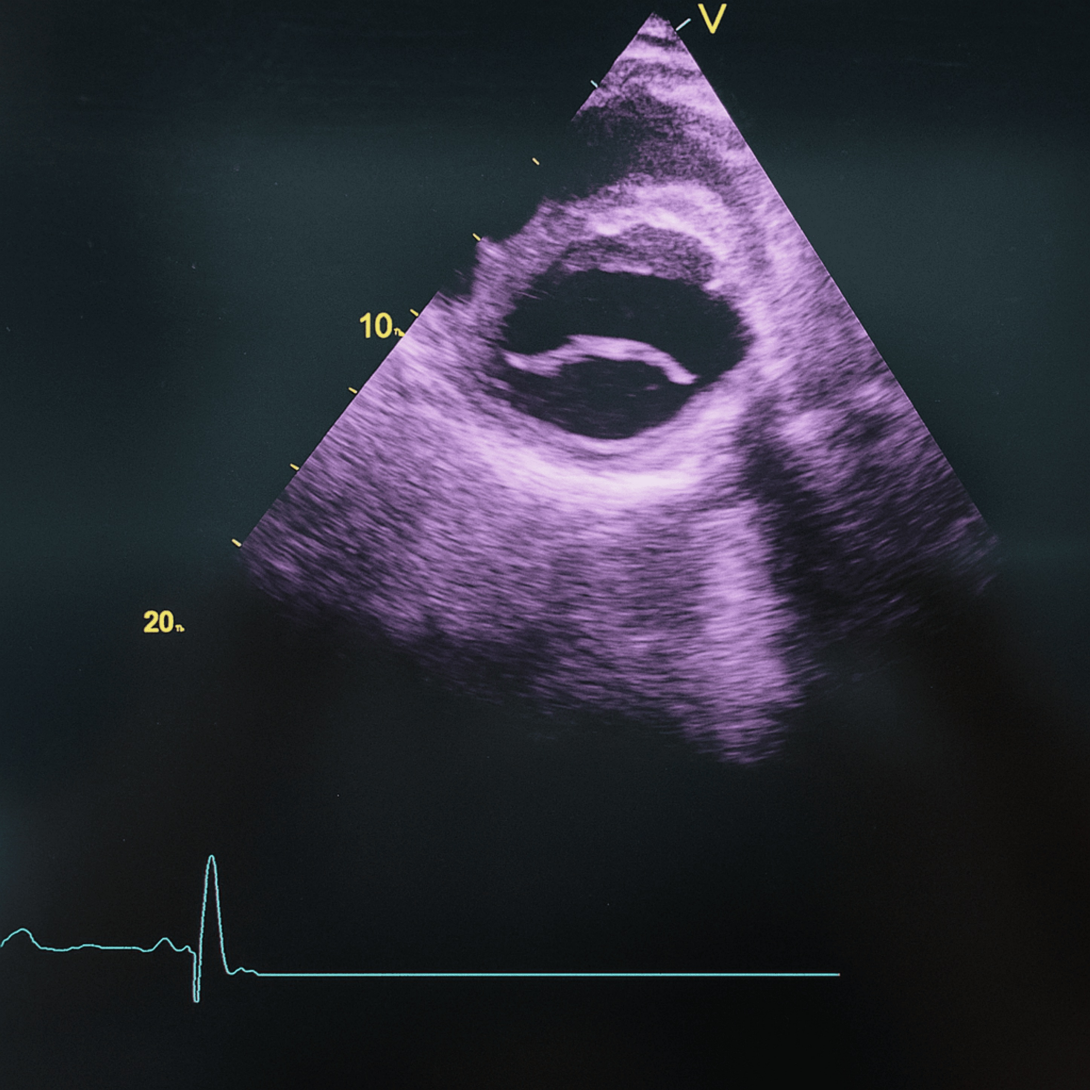
Echocardiography showing a bicuspid aortic valve

The echocardiography of the patient showed a bicuspid aortic valve with left ventricular hypertrophy and diastolic dysfunction (first degree) (Fig. 5).

During the hospital stay, the patient was treated with omeprazole 40 mg IV BID, perindopril/amlodipin 5/5 BID, and Ringer's 500 ml was used for hydration and potassium replacement. The patient was discharged in improved condition with further gastroprotective and antihypertensive therapy.

## Discussion

Hypothesis of a concomitant Klinefelter syndrome in a man with focal dermal hypoplasia (FDH) and mosaicism

Goltz syndrome, also known as focal dermal hypoplasia (FDH), is an extremely rare genetic disorder with a prevalence of one case per one million, characterized by specific cutaneous manifestations and a wide range of anomalies affecting the eyes, teeth, skeleton, urinary, digestive, cardiovascular, and central nervous systems [4]. In 1962, Goltz et al. described three patients with a significant congenital deficiency of connective tissue in the skin with the name focal dermal hypoplasia [5]. However, cases with similar findings had been described earlier with the name atrophoderma linearis maculosa et papillomatosis congenitalis [6]. Males with this condition do not usually survive, as the X-linked dominant mutation in the *PORCN *gene is usually lethal to XY individuals in utero, due to the lack of a second normal X-chromosomal allele to compensate. The patient in the presented case is a 43-year-old man diagnosed with focal dermal hypoplasia, which in itself suggests an exception to the usual genetic logic of the disease. This provides grounds for the hypothesis that the man does not have a typical XY karyotype, but most likely has Klinefelter syndrome (47,XXY), which would explain both the presence of an additional X-chromosomal allele and the possibility of survival with a *PORCN *mutation. In XXY individuals, the presence of two X chromosomes is observed, one of which can carry the *PORCN *mutation and the other normal, which allows for mosaicism or partial expression, analogous to the female phenotype in FDH [7]. Usually, in females, one X chromosome is inactivated (Barr body). The same could happen in a patient with Klinefelter syndrome. If more than one X chromosome with a mutation in the *PORCN *gene is inactivated, this would lead to a milder clinical picture. Karyotyping (Giemsa-trypsin-Giemsa (GTG) banding) or other genomic methods (e.g., array comparative genomic hybridization (aCGH), fluorescence in situ hybridization (FISH), or next-generation sequencing (NGS)) can be performed to confirm the presence of Klinefelter syndrome. In this case, the relatives of the patient refused further genetic counseling to prove this hypothesis. Only one other patient (female) is reported worldwide with Gotlz syndrome and a bicuspid aortic valve [8]. 

Functionally atypical expression of the X chromosome

Epigenetics is a set of heritable and reversible molecular mechanisms that regulate gene expression without altering the primary DNA sequence. These mechanisms are essential for normal development, cell differentiation, and tissue-specific regulation of gene activity. The main epigenetic processes include DNA methylation, posttranslational modifications of histones, and regulation by non-coding RNAs. Although males have only one X chromosome, epigenetic modifications (e.g., promoter methylation) can partially suppress expression of the mutant allele if the mutation is not fully active or if there is additional regulatory control on *PORCN*. This may lead to reduced expression of the mutated gene, similar to compensation [9].

## Conclusions

Encountering patients with Goltz syndrome (GDS) is rare but not impossible. This genetic syndrome affects many different organs and systems and may have different manifestations due to the superimposition of other genetic variants in individual patients. In this study, we also emphasize the importance of a detailed systemic examination to detect primary organ involvement. In-depth knowledge of the syndrome and the possibility of variations of its clinical picture in individuals due to personal characteristics in each genome is essential for the proper treatment of patients. Patients require a multidisciplinary approach and complex treatment for longer survival. Genetic testing is the gold standard for diagnosis, but it is too expensive, so clinicians should be aware of the clinical manifestations for diagnosis, so these patients are able to receive better care. Male patients can have complications related to gastrointestinal bleeding, metabolic syndrome, and arterial hypertension.
